# Integrated transcriptomics, proteomics and metabolomics-based analysis uncover TAM2-associated glycolysis and pyruvate metabolic remodeling in pancreatic cancer

**DOI:** 10.3389/fimmu.2023.1170223

**Published:** 2023-08-17

**Authors:** Xin Li, Yan Du, Wenkai Jiang, Shi Dong, Wancheng Li, Huan Tang, Jianfeng Yi, Wence Zhou, Hui Zhang

**Affiliations:** ^1^ Department of General Surgery, Lanzhou University Second Hospital, Lanzhou, China; ^2^ The Second School of Clinical Medicine, Lanzhou University, Lanzhou, China; ^3^ Department of General Surgery, The First School of Clinical Medicine of Lanzhou University, Lanzhou, China; ^4^ Department of Surgery, The First School of Clinical Medicine of Gansu University of Chinese Medicine, Lanzhou, China

**Keywords:** pancreatic cancer, tumor-associated macrophage 2, glycolysis, pyruvate, metabolic classification

## Abstract

**Introduction:**

Tumor-associated macrophage 2 (TAM2) abundantly infiltrates pancreatic ductal adenocarcinoma (PAAD), and its interaction with malignant cells is involved in the regulation of tumor metabolism. In this study, we explored the metabolic heterogeneity involved in TAM2 by constructing TAM2-associated metabolic subtypes in PAAD.

**Materials and methods:**

PAAD samples were classified into molecular subtypes with different metabolic characteristics based on a multi-omics analysis strategy. 20 PAAD tissues and 10 normal pancreatic tissues were collected for proteomic and metabolomic analyses. RNA sequencing data from the TCGA-PAAD cohort were used for transcriptomic analyses. Immunohistochemistry was used to assess TAM2 infiltration in PAAD tissues.

**Results:**

The results of transcriptomics and immunohistochemistry showed that TAM2 infiltration levels were upregulated in PAAD and were associated with poor patient prognosis. The results of proteomics and metabolomics indicated that multiple metabolic processes were aberrantly regulated in PAAD and that this dysregulation was linked to the level of TAM2 infiltration. WGCNA confirmed pyruvate and glycolysis/gluconeogenesis as co-expressed metabolic pathways of TAM2 in PAAD. Based on transcriptomic data, we classified the PAAD samples into four TAM2-associated metabolic subtypes (quiescent, pyruvate, glycolysis/gluconeogenesis and mixed). Metabolic subtypes were each characterized in terms of clinical prognosis, tumor microenvironment, immune cell infiltration, chemotherapeutic drug sensitivity, and functional mechanisms.

**Conclusion:**

Our study confirmed that the metabolic remodeling of pyruvate and glycolysis/gluconeogenesis in PAAD was closely related to TAM2. Molecular subtypes based on TAM2-associated metabolic pathways provided new insights into prognosis prediction and therapy for PAAD patients.

## 1 Introduction

Tumor progression and development require the metabolic reprogramming of cancer cells (1). Changes in the energy metabolism pattern of cancer cells can meet the needs of rapid proliferation and adaptation to the tumor microenvironment (2). Metabolic status in the tumor microenvironment (TME) is influenced by many factors, including angiogenesis, interactions with other cells, and systemic metabolism (3). Metabolic heterogeneity can influence therapeutic effectiveness and may predict clinical outcomes (4). However, few studies have focused on methods for distinguishing subtypes based on metabolic heterogeneity.

Pancreatic cancer (PAAD) is a malignancy with poor prognosis and high mortality (5). Tumor-associated macrophages (TAMs) abundant infiltration is a prominent characteristic of PAAD (6). TAMs can interact with pancreatic cancer cells to regulate metabolic, inflammatory, and immune states, forming an immunosuppressive TME and ultimately promoting tumor occurrence and development (7). Studies have demonstrated macrophages impact the biological function of pancreatic cancer cells, such as glucose metabolism (8, 9). However, a method that combines TAM- and metabolism-related characteristics to identify new molecular subtypes of PAAD remains lacking. Metabolic subtypes offer a unique vision for classifying patients for new generation cancer treatment; thus, developing new molecular subtypes according to immune-metabolic characteristics is crucial to improve precision medicine.

Herein, we classified PAAD into four metabolic subtypes with distinct immune-metabolic characteristics according to multi-omics and explored the function of detected metabolic subtypes in the clinical features, prognosis, and treatment of PAAD, aiming to provide a new understanding of immunotherapy for PAAD.

## 2 Materials and methods

### 2.1 Patients and samples

In this study, 30 pancreatic tissues were retrieved, including 20 samples of pancreatic ductal cell carcinoma and 10 of adjacent healthy tissues. All pancreatic samples were obtained from the First Hospital of Lanzhou University from January 2016 to December 2020. M2 macrophage markers (CD206 and CD163) were quantitatively assessed by immunohistochemical (IHC) staining in all samples, and the **Table 1** shows the features of these patients. All samples were acquired during the surgical procedures and were diagnosed with pancreatic ductal adenocarcinoma via postoperative pathology. The exclusion criteria were as follow: (I) comorbidity with other cancers; (II) metabolic illnesses; and (III) underwent preoperative chemotherapy or radiotherapy. The research followed the Declaration of Helsinki, and was approved by the Ethics Committee of First Hospital of Lanzhou University (No: LDYYLL2022-196).

**Table 1:** Clinical characteristics of 20 PAAD patients.

Characteristics	CD206 expression			CD163 expression		
	High (n=10)	Low (n=10)	*P* value	High (n=10)	Low (n=10)	*P* value
Age						
<60	4(50%)	4(50%)	1	5(62.5%)	3(37.5%)	0.65
>60	6(50%)	6(50%)		5(41.7%)	7(58.3%)	
Gender						
Female	6(60%)	4(40%)	0.371	6(60%)	4(40%)	0.371
Male	4(40%)	6(60%)		4(40%)	6(60%)	
CA199						
<=35	1(20%)	4(80%)	0.303	2(40%)	3(60%)	1
>35	9(60%)	6(40%)		8(53.3%)	7(46.7%)	
Histologic grade						
G3	7(63.6%)	4(36.4%)	0.37	8(72.7%)	3(27.3%)	0.07
G2	3(33.3%)	6(66.7%)		2(22.2%)	7(77.8%)	
Pathologic stage						
Stage1	0(0%)	7(100%)	0.003	3(42.9%)	4(57.1%)	1
Stage2/3	10(76.9%)	3(23.1%)		7(53.8%)	6(46.2%)	
T stage						
T1	3(75%)	1(25%)	0.582	2(50%)	2(50%)	1
T2/3	7(43.8%)	9(56.3%)		8(50%)	8(50%)	
N stage						
N0	0(0%)	7(100%)	0.003	3(42.9%)	4(57.1%)	1
N1	10(76.9%)	3(23.1%)		7(53.8%)	6(46.2%)	
Vascular invasion						
No	6(50%)	6(50%)	1	7(58.3%)	5(41.7%)	0.65
Yes	4(50%)	4(50%)		3(37.5%)	5(62.5%)	
M stage						
M0	10(50%)	10(50%)		10(50%)	10(50%)	

### 2.2 RNA-Seq data download

The Cancer Genome Atlas (TCGA) and The Genotype-Tissue Expression (GTEx) contain a total of 178 PAAD and 171 healthy pancreatic tissues (10, 11). The University of California Santa Cruz (UCSC) database collates and standardizes the high-throughput sequencing (HTSeq) data in TCGA and GTEx, which can be directly obtained for differential expression analysis of genes (12). TCGA-PAAD, GTEx- pancreas and TCGA Pan-Cancer data were obtained from UCSC on July 1, 2022, all in transcripts per million formats (TPM). The clinical information of PAAD patients was obtained from TCGA. After deleting samples with missing survival data, a total of 178 patients were included. In addition, PAAD microarray data from Gene Expression Omnibus (GEO, GSE15471, GSE62165, GSE62452) were downloaded for this study (13).

### 2.3 Proteomics and targeted metabolomics assays

In proteomics assays, the EasynLC1200 chromatography system (Thermo Scientific) was used to perform chromatographic separations. Buffer consists of two liquid aqueous solutions: A is 0.1% formic acid, and B is 0.1% formic acid acetonitrile (acetonitrile is 85%). DDA (data-dependent acquisition) mass spectrometry was conducted using a Q-Exactive HF-X mass spectrometer (Thermo Scientific). All mass spectrometry data were merged by Proteome Discoverer 2.4 software to analyze the search library and build a spectral database. In targeted metabolomics assays, separation was performed by high-performance liquid chromatography using Shimadzu NexeraX2LC-30AD. Mobile phase: Liquid A was 5% aqueous acetonitrile, 10 mM ammonium acetate, pH 9; Liquid B was 95% aqueous acetonitrile, 10 mM ammonium acetate, pH 9. The sample was injected into the autosampler at a column temperature of 40°C, a flow rate of 300 µL/min, and a sample volume of 8 µL. Mass spectrometry was conducted utilizing a QTRAP 5500 mass spectrometer (ABSCIEX) in positive/negative ion mode. To determine the ion pair that needed to be monitored, MRM mode was employed.

### 2.4 Proteomics and metabolomics analysis

After obtaining the protein identification matrix, proteins with more missing values in the matrix were filtered out by the 50% principle. The Limma R software was employed to carry out difference analysis, and differentially expressed proteins (DEPs) were defined as those with a P value < 0.05 and an absolute value of log fold change (LogFC) > 1 (14). R tool “ClusterProfiler” was utilized to assess the KEGG pathways involved in DEPs (15). Using MultiQuant program, chromatographic peak regions and retention periods were retrieved for targeted metabolomics sequencing. Metabolite identification was performed using corrected retention times of energy metabolite standards. Forty energy metabolites were quantified in all samples, and the screening criteria for differentially expressed metabolites (DEMs) was a T test P value < 0.05. The MetaboAnalyst 5.0 database was utilized to conduct KEGG enrichment analysis of DEMs (16).

### 2.5 TAM2-related metabolic pathway analysis

To analyze TAM2-associated protein functions, co-expressed proteins of CD206 and CD163 were obtained by Pearson correlation analysis, and the screening criteria were defined as a P value < 0.05 and a correlation coefficient >0.3. KEGG enrichment analysis was carried out to assess the biological processes involved in TAM2-associated proteins. Given the important role TAM2 plays in the metabolic remodeling of PAAD, we further integrated metabolomics and IHC data to identify metabolite modules closely associated with TAM2 infiltration levels by weighted gene co-expression network analysis (WGCNA). All recognized metabolites were included in the construction of the co-expression network by the WGCNA R package (17). The samples with a large dispersion are removed by hierarchical clustering. The soft power of k = 6 was selected. Subsequently, the transformation of the expression matrix into a topology matrix was executed. The hybrid dynamic shearing tree standard was employed to cluster genes using the average-linkage hierarchical clustering approach, according to TOM. The module trait correlation was based on the module and the TAM2 infiltration level determined by Pearson’s relevant tests, and the metabolites contained in the module were defined as TAM2-associated metabolites when the P value was ≤0.05. Finally, the metabolic pathways involved in TAM2 were identified based on TAM2-associated metabolites in the MetaboAnalyst 5.0 database.

### 2.6 Metabolic subtype classification

The metabolic subtypes of PAAD were further investigated based on TCGA-PAAD transcriptome data. Genes from the gene sets hsa00620 and hsa00010 of the Molecular Signatures Database (MSigDB) were utilized as pyruvate and glycolysis/gluconeogenesis (GG) metabolism-associated genes, respectively (18). Hierarchical clustering was conducted on pyruvate and GG metabolism-associated genes utilizing the HCLUST R function, and genes with dispersed expression in pyruvate and GG metabolism-associated genes were excluded. To categorize samples into the quiescent (pyruvate ≤ 0, GG ≤ 0), pyruvate (pyruvate > 0, GG 0), GG (pyruvate ≤ 0, GG > 0), and mixed (pyruvate > 0, GG > 0) metabolic subtypes, the median expression patterns of pyruvate and GG metabolism-associated genes were employed. To assess the reliability of the metabolic subtypes, the expression of pyruvate pathway genes, GG pathway genes and TAM2-associated genes was compared among different subtypes. The design style of integrating a multi-omics strategy for metabolic subtype classification was informed by previous studies (19).

### 2.7 Clinical features and mechanistic investigation of metabolic subtype

Kaplan-Meier survival curves were created to explore variations in survival (overall survival [OS], disease-specific survival [DSS], progression-free survival [PFS]) among various metabolic subtypes utilizing the R tools Survival and Survminer (20). The clinical characteristics of the different metabolic subtypes were also examined by chi-square or Fisher’s exact test. Characteristic molecules for each metabolic subtype were obtained by differential analysis using the Limma R package and in accordance with the following screening criteria: 1. genes showed significant upregulation in one subtype in comparison with the other three subtypes; 2. P value < 0.05 and LogFC > 0.6. Using Cytoscape plugins ClueGO and ClusterProfiler R packages, the enriched pathways were detected by these characteristic molecules to evaluate probable pathway deficits across several metabolic subtypes (21). The parameters for the ClueGO plugin were set as follows: the ontologies were set to include biological process, cellular component, and molecular function; only pathways with a p-value less than 0.05 were displayed; The GO tree interval was set to range from 3 to 8, and the Kappa score is set to 0.5. The parameters for the ClusterProfiler R packages were set as follows: organism = hsa, pvalueCutoff = 0.05, pAdjustMethod = fdr, qvalue Cutoff = 1.

### 2.8 Immune infiltration of metabolic subtypes

The composition of the tumor microenvironment for all TCGA-PAAD samples was calculated by the Estimate R package, such as immune, stromal and estimate scores. Subsequently, the Wilcox test was employed to detect significant differences in microenvironmental scores among distinct metabolic subtypes. Additionally, a thorough investigation of the infiltration intensity of each kind of immune cell was conducted utilizing a variety of algorithms such as TIMER, CIBERSORT, xCELL, EPIC, MCPcounter, and QUANTIseq Each sample’s immune cell infiltration by various metabolic subtypes was shown using the Heatmap R tool, and Kruskal test results were used to detect significant differences. Additionally, we analyzed the differential expression of immunosuppressive gene sets in different metabolic subtypes by the Wilcox test.

### 2.9 Therapeutic prediction of metabolic subtype

Considering the application of chemotherapeutic agents and immune checkpoint inhibitors in PAAD, we analyzed the response of patients with different metabolic subtypes to targeted therapy. Somatic mutation data of TCGA-PAAD were integrated and analyzed by the Maftools R package, and chi-square test was employed to calculate significant variations (22). According to their gene expression patterns, the “Oncopredict” R tool was employed to calculate each TCGA-PAAD patient’s treatment sensitivity (23). The parameters for the “Oncopredict” R tool were set as follows: trainingPtype = GDSC2_Res, testExprData = testExpr, batchCorrect = eb, powerTransformPhenotype = TRUE, removeLowVaryingGenes = 0.2, minNumSamples = 10, printOutput = TRUE, removeLowVaringGenesFrom = rawData. The estimated drugs include common clinical agents and clinical trial agents for pancreatic cancer. Subsequently, the Wilcox test was utilized to examine the four metabolic subtypes for medicines that could be sensitive, with lower half maximal inhibitory concentration (IC50) measures revealing higher drug sensitivity.

### 2.10 Statistical analysis

Software used for statistical analysis of data: R (version 4.0.2), SPSS (version 25.0), Prism (version 8.0.2). Test methods for calculating significant differences included the Kruskal test, Wilcox test and chi-square test. Correlation analysis using Pearson’s test. Differential analysis of expression profiles using the Limma R package. Tools used for data visualization: ggplot2 R package, Prism (version 8.0.2).

## 3 Results

### 3.1 TAM2 is highly infiltrative in PAAD and associated with poor prognosis

**Figure 1:**
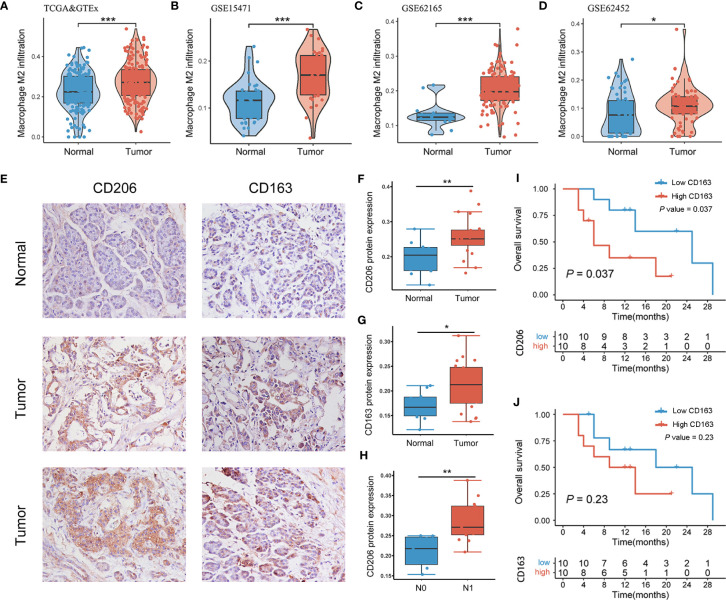
TAM2 infiltration was abnormally elevated in PAAD. TAM2 infiltration in PAAD and healthy pancreatic tissues was calculated by the CIBERSORT algorithm in TCGA&GTEx **(A)**, GSE15471 **(B)**, GSE62165 **(C)** and GSE62452 **(D)**. **(E)** Immunohistochemical staining of CD206 and CD163 in PAAD tissue and normal pancreatic tissue. Quantitative analysis of CD206 **(F)** and CD163 **(G)** proteins in PAAD and healthy pancreatic tissues. **(H)** Differential expression of CD206 protein in PAAD patients with N0 and N1 stages. Survival analysis of CD206 **(I)** and CD163 **(J)** proteins in 20 patients with PAAD. TAM2, tumor-associated macrophage 2; PAAD, pancreatic cancer; TCGA, The Cancer Genome Atlas; GTEx, genotype-tissue expression. *P < 0.05, **P < 0.01, ***P < 0.001.

Integrating TCGA-PAAD and GTEx-pancreas data, the TAM2 infiltration level of all samples was measured using CIBERSORT algorithm. The findings revealed significant upregulation of TAM2 infiltration in PAAD than pancreatic tissues (**Figure 1A**). Microarray data sourced from the GEO database were used to validate this result. In GSE15471, GSE62165 and GSE62452, TAM2 infiltration in PAAD tissue was abnormally abundant (**Figures 1B–D**). CD206 and CD163 are the classical cellular markers of TAM2. Pan-cancer analysis of the TCGA&GTEx database showed that CD206 and CD163 mRNA expression was heterogeneous among 33 malignant tumor tissues, but both were significantly highly expressed in PAAD tissues (**Figures S1A, B**; **Table S1**). Subsequently, we quantified the protein expression of CD206 and CD163 by IHC staining in 20 PAAD tissues and 10 normal pancreatic tissues (**Figure 1E**). Consistent with the mRNA levels, the results indicated that CD206 and CD163 protein expression was upregulated in PAAD (**Figures 1F–H**; **Table S2**). Moreover, survival analysis findings indicated that PAAD patients in the high CD206 or CD163 protein expression groups exhibited shorter survival times compared to the low expression group, although this difference was not statistically significant for CD163 (**Figures 1I, J**). These results suggest that TAM2 is abundantly infiltrated in the PAAD microenvironment and may be associated with the malignant progression of the tumor.

### 3.2 Proteomics and metabolism suggest metabolic abnormalities in pancreatic cancer

**Figure 2:**
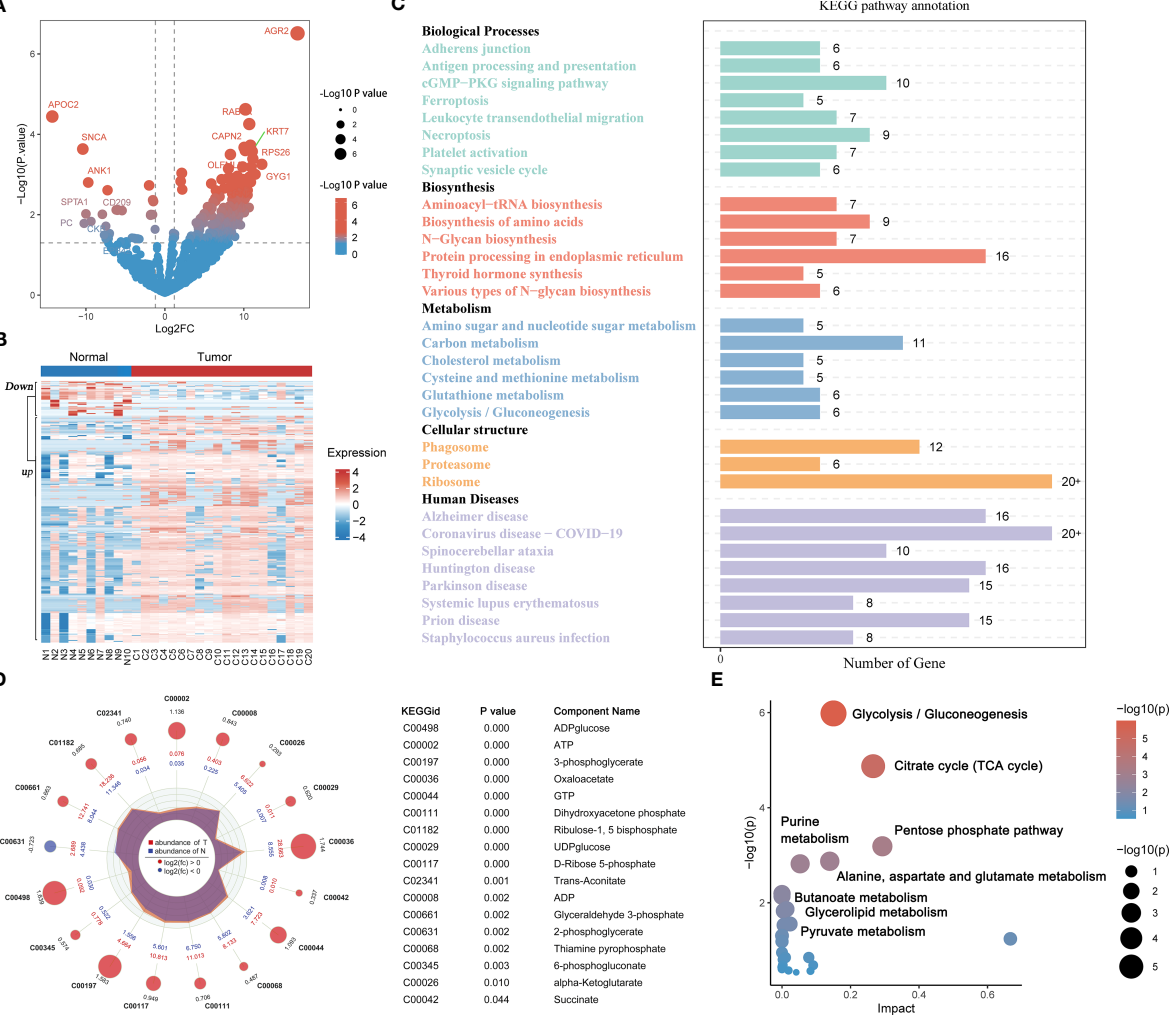
Proteomics and metabolomics analysis in PAAD and healthy pancreatic tissues. Volcano **(A)** and heatmaps **(B)** of differentially expressed proteins in 20 PAAD tissues and 10 normal pancreatic tissues. **(C)** KEGG functional enrichment analysis for differentially expressed proteins. **(D)** Radar plot of differentially expressed metabolites in 20 PAAD tissues and 10 normal pancreatic tissues. **(E)** KEGG functional enrichment analysis for differentially expressed metabolites. PAAD, pancreatic cancer; KEGG, Kyoto Encyclopedia of Genes and Genomes.

Differential analysis based on the proteomic matrix screened a total of 310 DEPs, including 269 upregulated DEPs and 41 downregulated DEPs in PAAD tissues (**Figure 2A**; **Table S3**). Hierarchical clustering plots showed that DEPs had different expression patterns in the normal pancreas group and PAAD group (**Figure 2B**). KEGG enrichment analysis findings revealed that the cellular pathways related to the DEPs included the following five main functional categories: biological processes, biosynthesis, metabolism, cellular structure and human diseases (P < 0.05, **Figure 2C**; **Table S4**). The metabolic pathways of the DEPs showed a correlation with glucose, lipid and amino acid metabolisms (**Figure 2C**). Targeted metabolomics analysis identified 40 energy metabolites, of which 16 metabolites were highly expressed in PAAD tissues. Log2FC, P value and expression level of DEMs are shown in a radar chart (**Figure 2D**). Functional enrichment analysis of the MetaboAnalyst 5.0 database revealed that DEMs have role in metabolic pathways, such as pyruvate metabolism, glycolysis/gluconeogenesis and the citrate cycle (TCA cycle) (P < 0.05, **Figures 2E**, **S2**; **Table S5**).

### 3.3 TAM2 is associated with metabolic abnormalities in PAAD

**Figure 3:**
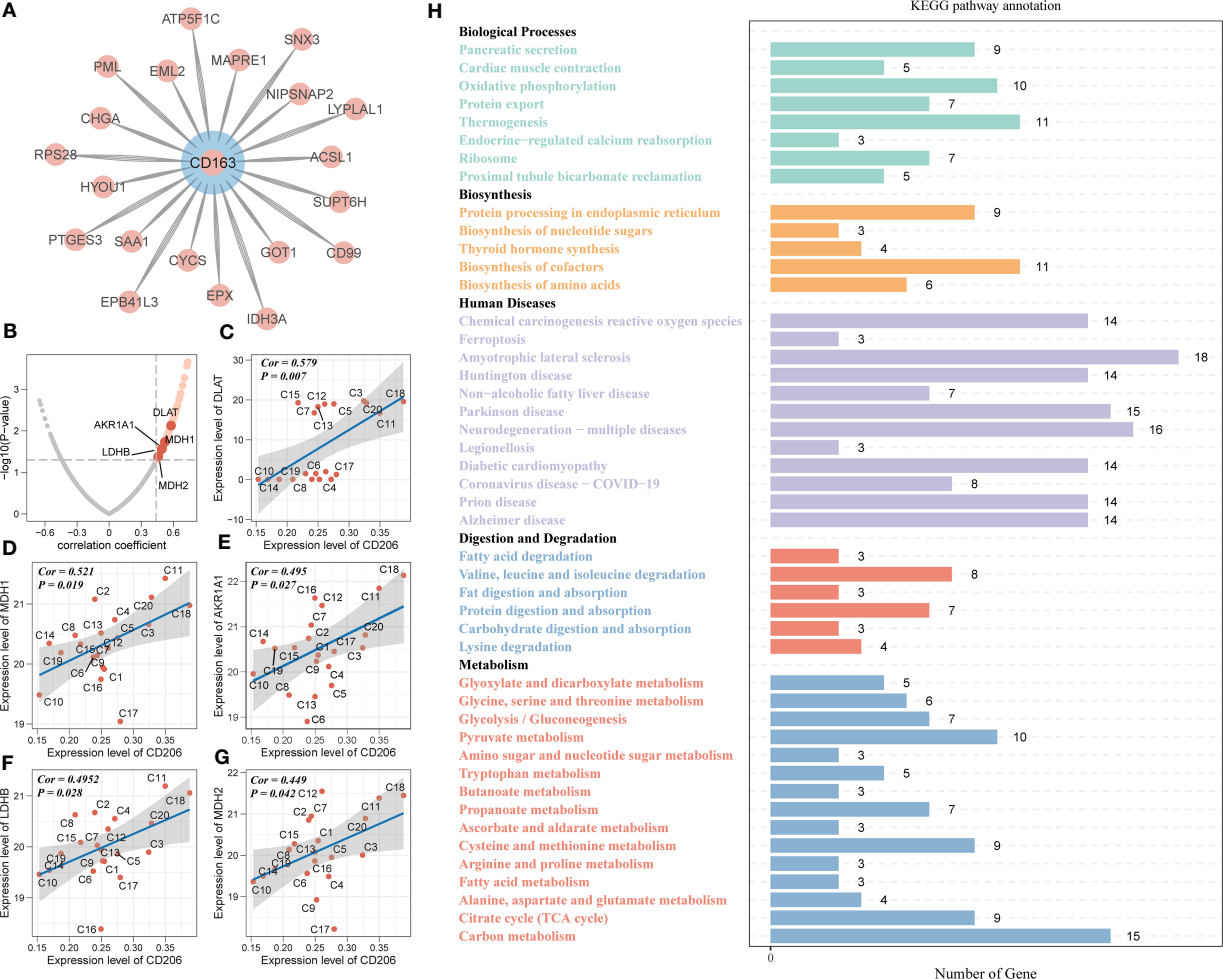
TAM2-related metabolomic analysis in PAAD. **(A)** Proteins co-expressed with the TAM2 cell markers CD163 **(A)** and CD206 **(B)**. Scatter plot of association of glycolysis- with pyruvate metabolism-related proteins **(C–G)** and CD206 expression. **(H)** KEGG functional enrichment analysis of KEGG for CD163 and CD206 co-expressed proteins. PAAD, pancreatic cancer; KEGG, Kyoto Encyclopedia of Genes and Genomes.

**Figure 4:**
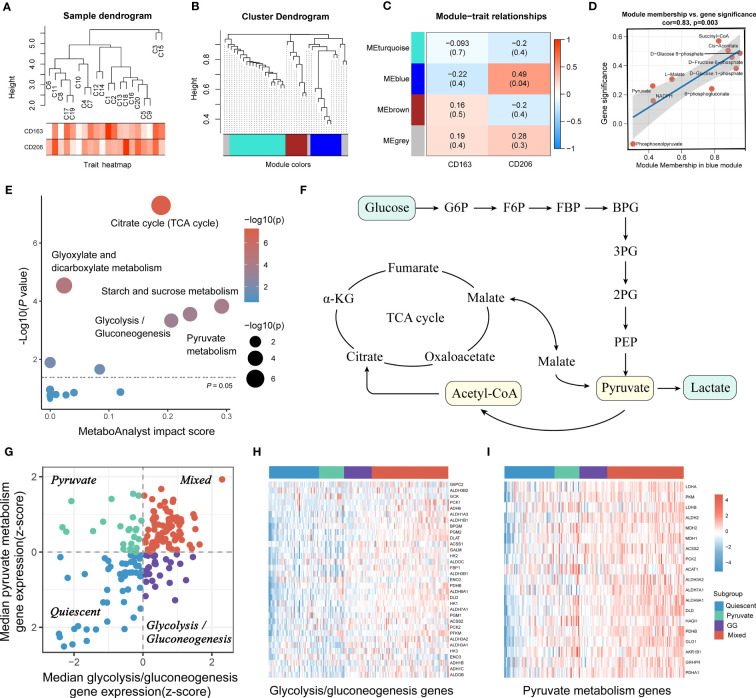
PAAD samples were classified into four subtypes based on TAM2-associated metabolic pathways. PAAD sample dendrogram **(A)**, metabolite cluster dendrogram **(B)**, correlation between metabolite module and TAM2 cell markers **(C)**, correlation between gene significance and module membership **(D)** of weighted metabolite co-expression network analysis based on metabolomics. **(E)** KEGG functional enrichment analysis for TAM2-related metabolite modules. **(F)** Crosstalk between pyruvate and GG metabolism. **(G)** The scatter plot demonstrates the metabolic isoforms based on TAM2-related metabolic pathways. The X and Y axes represent the median expression patterns of GG metabolism-associated genes and pyruvate metabolism-related genes in PAAD samples, respectively. Heatmap showing the expression of GG metabolism-associated genes **(H)** and pyruvate metabolism-related genes **(I)** in four metabolic subtypes. PAAD, pancreatic cancer; TAM2, tumor-associated macrophage 2; KEGG, Kyoto Encyclopedia of Genes and Genomes; GG, glycolysis/gluconeogenesis.

Based on the proteomic, metabolomic and immunohistochemical data of 20 PAAD tissues, we further analyzed the biological mechanisms involved in tumor metabolism by TAM2. A total of 20 CD163 co-expressed proteins and 155 CD206 co-expressed proteins were screened by Pearson correlation analysis (P value < 0.05 and correlation coefficient >0.3, **Figures 3A, B**; **Table S6**). AKR1A1, LDHB, MDH1/2 and DLAT were closely associated with glycolysis and pyruvate metabolism and exhibited a significant positive correlation with CD206 in PAAD (**Figures 3C–G**). According to KEGG pathway analysis findings, the functions of CD206 and CD163-related proteins were mainly enriched in five biological directions, with the metabolic pathway accounting for the largest proportion (**Figure 3H**; **Table S7**). We further analyzed the TAM2-associated metabolite module by WGCNA. Samples C3 and C15 were dispersed from the other samples in the sample dendrogram and were excluded from the WGCNA (**Figure 4A**). All metabolites were divided into four modules according to the association of expression in the cluster dendrogram (**Figure 4B**). The correlation between each metabolite module and CD206 or CD163 was examined using Pearson’s test. Consequently, findings illustrated close association between the blue metabolite module with CD206 expression (**Figure 4C**). The gene significance was highly significantly related to module membership in the blue module (**Figure 4D**). KEGG enrichment analysis of the metabolites contained in the blue module showed that starch and sucrose metabolism and GG and pyruvate metabolism had high pathway impact scores (P value < 0.05 and impact scores>0.2, **Figure 4E**). These results suggested that TAM2 was closely associated with the metabolic remodeling of PAAD and mainly involved in GG and pyruvate metabolism.

### 3.4 Detection of four metabolic subgroups of PAAD according to GG and pyruvate metabolism-related genes

**Figure 5:**
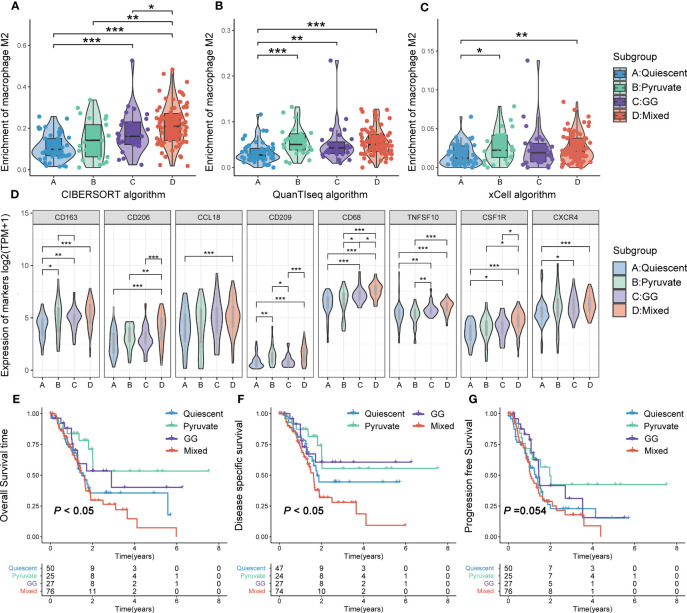
TAM2 infiltration and survival analysis of metabolic subtypes. Comparison of TAM2 infiltration levels in different metabolic subtypes using the CIBERSORT **(A)**, xCELL **(B)** and QUANTIseq **(C)** algorithms. **(D)** Differential expression of TAM2 cellular markers from the Cellmarker database in metabolic subtypes. Overall survival **(E)**, disease-specific survival **(F)**, and progression-free survival **(G)** Kaplan–Meier curves of PAAD patients with different metabolic subtypes. TAM2, tumor-associated macrophage 2; PAAD, pancreatic cancer. *P < 0.05, **P < 0.01, ***P < 0.01.

RNA-HTSeq data from the TCGA-PAAD cohort were used to classify PAAD according to the expression profiles of pyruvate and GG metabolism-associated genes. All PAAD samples were classified into the following four subgroups: quiescent, pyruvate, GG and mixed (**Figures 4F, G**). The heatmap demonstrated that the expression of pyruvate and GG metabolism-associated genes sequentially increased from the quiescent subgroup to the mixed subgroup (**Figures 4H, I**, **S3**). CIBERSORT, xCELL as well as QUANTIseq algorithms were utilized to calculate the infiltration level of TAM2 infiltration in each subtype. The three algorithms yielded consistent results showing the highest TAM2 infiltration levels for the mixed subtype, the lowest for the quiescent subtype, and no significant difference between the pyruvate and GG subtypes (**Figures 5A–C**). The expression levels of TAM2 markers from the Cellmarker database also increased gradually from the quiescent subtype to the mixed subtype (**Figure 5D**). Survival analysis of OS, PFS and DSS suggested a significant difference of the outcome among the four metabolic subtypes, which in turn revealed clinical significance among the classification of metabolic subtypes (**Figures 5E–G**). Moreover, the analysis of clinical characteristics findings illustrated that only T-stage differed among the different metabolic subtypes, suggesting a higher proportion of the mixed subgroup in PAAD patients with T3/4 stage (**Figure S4**).

### 3.5 Different metabolic subtypes were involved in different biological mechanisms

**Figure 6:**
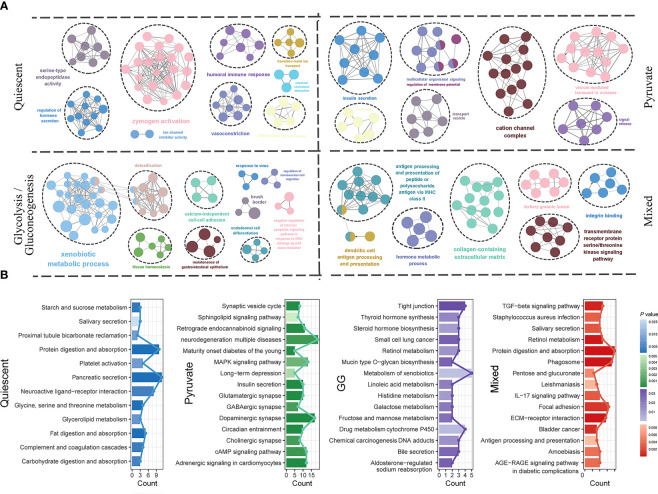
Functional mechanism characteristics of metabolic subtypes. GO functional annotation **(A)** and KEGG pathway annotation **(B)** for molecular characteristics of quiescent, pyruvate, glycolysis/gluconeogenesis and mixed metabolic subtypes.GO, Gene Ontology; KEGG, Kyoto Encyclopedia of Genes and Genomes; PAAD, pancreatic cancer.

The characteristic genes of each subtype were obtained by differential analysis to represent the molecular characteristics of the corresponding subgroup. The quiescent subtype contained 104 characteristic genes, the pyruvate subtype contained 364 characteristic genes, the GG subtype contained 147 characteristic genes, and the mixed subtype contained 151 characteristic genes (**Table S8**). The functional annotation of characteristic genes of distinct metabolic subtypes differed significantly. For GO terms, serine-type endopeptidase activity, hormone secretion, zymogen activation and immune response was characteristics for the quiescent subtype; the pyruvate subtype was characterized by cation channel complex, vesicle-mediated transport and insulin secretion; the GG subtype was characterized by xenobiotic metabolic process, detoxification, and tissue homeostasis; and the mixed subtype was characterized by extracellular matrix, antigen presentation and serine/threonine kinase signaling pathway (**Figure 6A**; **Table S9**). KEGG pathways of the quiescent subtype were dominated by glucose, amino acid, and lipid metabolisms. KEGG pathways of the pyruvate subtype were closely related to the MAPK and CAMP signaling pathways. KEGG pathways of the GG subtype were enriched in substance synthesis and glucose metabolism. KEGG pathways of the mixed subtype were involved in immune-related biological processes and signaling molecules (**Figure 6B**; **Table S10**). These results suggested that the function of metabolic subtypes was not only complex but also distinctive in the different subtypes.

### 3.6 Immune infiltration analysis, immunotherapy prediction and targeted drugs sensitivity analysis

**Figure 7:**
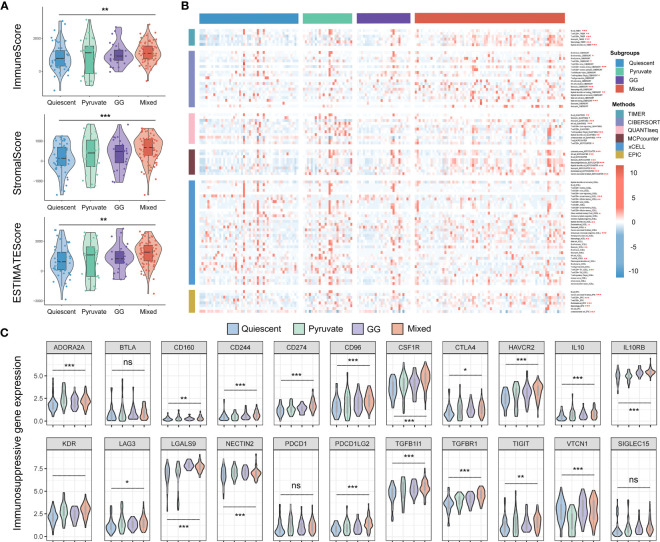
Immune microenvironment analysis of metabolic subtypes. **(A)** The Estimate method was utilized to compare the immune, stromal, and ESTIMATE scores across metabolic types in the TCGA-PAAD cohort. **(B)** Distribution of immune cell infiltration among the four metabolic subtypes according to the TIMER, CIBERSORT, xCELL, EPIC, MCPcounter and QUANTIseq methods. **(C)** Differential expression of immunosuppressive genes and immune checkpoint genes in different metabolic subtypes. TCGA, The Cancer Genome Atlas; PAAD, pancreatic cancer. *P < 0.05, **P < 0.01, ***P < 0.01.

**Figure 8:**
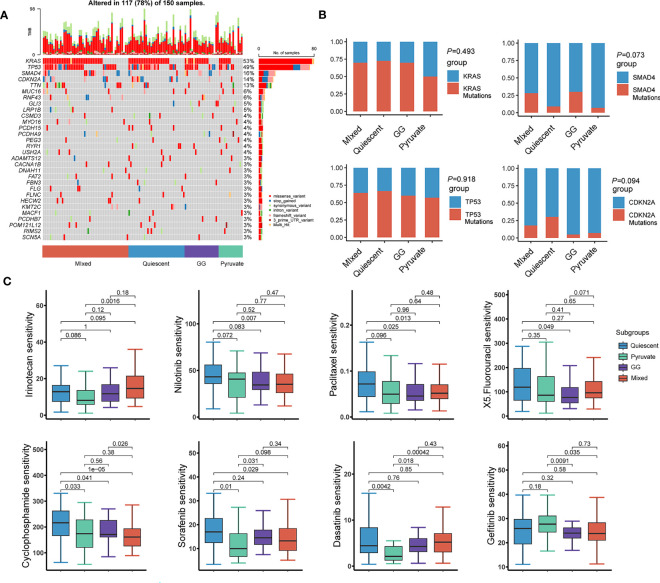
Chemotherapy sensitivity estimation and immunotherapy response analysis. **(A)** Waterfall diagram showing the genes with high mutation frequency in the somatic mutation data of TCGA-PAAD. **(B)** Comparison of mutation frequencies of highly mutated genes in different metabolic subtypes. **(C)** IC50 values of 8 chemotherapeutic agents in PAAD patients with different metabolic subtypes. TCGA, The Cancer Genome Atlas; PAAD, pancreatic cancer; IC50, half maximal inhibitory concentration.

Given that the immunity is essential constituent of the functional mechanisms of metabolic subtypes, we further analyzed immune cell infiltration in distinct metabolic subtypes. Immunoassay scores obtained utilizing the ESTIMATE method were significantly different across metabolic subtypes, indicating a relationship between metabolic subtypes categorization and tumor purity in PAAD samples (**Figure 7A**). The results of immune infiltration analysis based on multiple algorithms showed that the infiltration levels of multiple immune cells were significantly different in various metabolic subtypes, including memory B cells, B cells, cancer-associated fibroblasts, endothelial cells, eosinophils, mast cells, monocytes, myeloid dendritic cells, neutrophils, NK, CD4+ memory T, CD4+ T, CD8+ T, and regulatory T cells (**Figure 7B**). In addition, most immunosuppressive genes and immune checkpoint genes were differentially expressed in different metabolic subtypes (**Figure 7C**). Tumor mutational burden is believed to predict the effect of tumor immunotherapy, so somatic mutation data from the TCGA-PAAD dataset were extracted for analysis. The results showed that KRAS mutations were the most frequent in the four metabolic subtypes, but no significant variation existed in the mutation frequency of highly mutated genes in the different metabolic subtypes (**Figures 8A, B**). The effects of metabolic remodeling on the prediction of chemotherapy response are significant. Using the OncoPredict R program, the IC50 values of prominent chemotherapeutic drugs and targeted medicines were calculated for each PAAD sample. The pyruvate subtype had decreased IC50 scores for dasatinib, irinotecan, rapamycin, sorafenib and X5 fluorouracil (**Figure 8C**). GG subtype had decreased IC50 scores for gefitinib, lapatinib and paclitaxel (**Figure 8C**). The mixed subtype had lower IC50 values for cyclophosphamide and nilotinib (**Figure 8C**).

## 4 Discussion

Currently, the treatment of pancreatic cancer emphasizes precision treatment. Treating PC requires accurate molecular characteristics and targeted medicines. Research based on the tumor microenvironment is increasingly used to guide novel therapies for pancreatic cancer. In this study, we created an unique multi-omics integration technique based on glycolysis and pyruvate metabolic processes, combined with macrophage infiltration to elucidate the metabolic variability of PAAD and its therapeutic significance. This can help identify PAAD patients who respond to immunotherapy and targeted therapies.

Macrophages can induce tumor cell proliferation and metastasis through angiogenesis, immunosuppression, hypoxia induction and other pathways and are involved in many tumor-promoting outcomes in cancer (24). Huilin Ye et al. found that CCL18, which secreted by tumor-associated M2 macrophage (TAM2), promotes the progression of pancreatic cancer and activates the glycolytic metabolic pathway in tumor cells. Conversely, VCAM-1 in PAAD cells was able to produce more lactate by promoting aerobic glycolysis, thereby inducing the macrophages which from the tumor microenvironment to polarize toward M2 macrophages (8). These results suggest that TAM2 and tumor cells form a mutually reinforcing positive feedback loop in pancreatic cancer (8). In addition, lactic acid produced by glycolytic metabolism in PAAD cells can induce macrophages to express VEGF dependent on HIF-1α and promote the conversion of M0 macrophages to M2 macrophages, which in turn enhances aerobic glycolysis in PAAD (25). We first compared the degree of macrophage infiltration between pancreatic cancer patients and the normal population, found that macrophages showed high infiltration in pancreatic cancer tissues, and searched for macrophage-related signaling pathways for cancer occurrence and development, including metabolism-related pathways. To clarify the metabolic status of pancreatic cancer, we performed metabolic analysis on tissue samples from pancreatic cancer patients. The results showed the metabolic characteristics of PAAD progression and identified the processes most associated with the development of pancreatic cancer: glycolysis and pyruvate metabolism.

Since Warburg proposed the theory of cancer metabolism, Numerous investigations have highlighted the correlation between cancer cells and glucose metabolism (26). Cancer metabolism is characterized by a preference for glycolysis to produce energy in a way that does not rely on oxygen (27). Glycolysis is key for pancreatic cancer cells to maintain their biosynthesis and energy requirements, promoting tumor invasion, metastasis and drug resistance (28). The large amount of lactic acid produced by cancer cells through glycolysis leads to the polarization of TAMs toward the M2 phenotype in the PC microenvironment (29). In this study, we also found that the higher glycolytic activity subtype (GG) showed higher M2-type macrophage infiltration. This is consistent with the relationship between glycolysis and macrophages in pancreatic cancer. Thus, inhibition of glycolysis is a promising strategy for targeting pancreatic cancer. Research has shown that Chromebox protein homolog 3 (CBX-3) is a positive modulator of glycolysis in pancreatic cancer cells, and that blocking the CBX3-FBP1 signaling axis inhibits aerobic glycolysis, which suggests that this approach may be useful in the treatment of pancreatic cancer (30). Additionally, glycolysis activation in pancreatic cancer can be reversed by an mTOR inhibitor (RAD001), and RAD001 in combination with gemcitabine can promote the chemotherapy sensitivity of PAAD cells (28).

Pyruvate is an important molecule for human metabolism. Pyruvate is the end product of glycolysis and is eventually transported to the mitochondria to be involved in the tricarboxylic acid cycle (31). Some enzymes in the pyruvate metabolic pathway, such as pyruvate kinase (PK) and pyruvate carboxylase, have been studied in cancer (32, 33). For instance, PKM2 has been shown to be overexpressed in various cancers and can promote cancer cell proliferation and metastasis (34). Additionally, pyruvate carboxylase activity has a function in protecting cancer cells from oxidative damage and regulating lipid metabolism (34). For PAAD, the survival time was significantly reduced in high PKM2 expression patients compared to low PKM2 expression patients. Moreover, PKM2 expression was negatively related to the number of CD8+ cells in the tumor (35). However, targeting these enzymes could attenuate glycolysis and inhibit tumor proliferation (36). From our results, the “pyruvate phenotype” seems to be less sensitive to some anticancer drugs. Therefore, targeting pyruvate metabolism-related enzymes in the future may help improve drug resistance in PAAD patients.

Since the in-depth study of immune checkpoints on the surface of T cells, immune checkpoints, such as programmed death receptor 1 (PD-1), have become new therapeutic targets for various cancers (37). However, PAAD does not respond well to immunotherapy because the TME of PAAD is immunosuppressed (38). At present, drug therapy for PAAD has gradually shifted to combination therapy with targeted therapy and immune checkpoint inhibitors. For instance, combination therapy targeting CXCL12 and PD-L1 may have anticancer effects in PAAD (39). Our study provided a new molecular subtype for PAAD based on immune status and metabolic features. The combination of targeted therapy with immunotherapy for patients with different immune metabolic phenotypes appears to be feasible in the future. Pancreatic stellate cell-secreted factors promote both glycolysis and gemcitabine resistance in PAAD cells, while drug resistance can be reduced when glycolysis is inhibited (40). Additionally, mTOR inhibitors can alter the TME of PAAD through metabolic reprogramming, thereby promoting the efficacy of PD-L1 blockers in combination with gemcitabine (41). Therefore, this combination therapy, including immunotherapy, will become a new treatment strategy for PAAD patients in the future.

This study has some limitations. Although the molecular subtypes based on the TCGA-PAAD cohort have shown advantages in clinical prognosis, tumor microenvironment and treatment outcome prediction, the reliability of molecular subtypes needs to be further validated in a large amount of clinical treatment data. Our study reveals that glycolysis and pyruvate-related metabolic remodeling occurs in pancreatic cancer tissues, but such metabolic alterations are not an exclusive signature of tumor cells. Single-cell sequencing and spatial proteomics could help clarify this scientific question. In addition, future work should focus on more novel immune-metabolic characteristics of PAAD, including glucose metabolism, the anoxic microenvironment and amino acid metabolism. The relationships between metabolites and immune cells in the TME should be studied in depth. We also need to pay attention to the metabolic characteristics of immune cells: macrophages, B cells, and T cells.

## 5 Conclusions

Transcriptomic and immunohistochemical analyses confirmed that TAM2 infiltration levels were abnormally elevated in PAAD and were associated with poor patient prognosis. Proteomic analysis highlighted the strong correlation between TAM2 and metabolic remodeling in PAAD patients. Metabolomic analysis further confirmed the co-expression relationship between pyruvate and glycolysis/gluconeogenesis metabolism and TAM2 infiltration in PAAD. This study identifies four subtypes of PAAD patients having various metabolic features according to TAM2-related metabolic pathways. Higher levels of pyruvate, glycolysis/gluconeogenesis metabolism, and higher levels of TAM2 infiltration, poorer survival prognosis and higher immune suppression are characteristics of the mixed subtype. In addition, different metabolic subtypes have different functional mechanisms and differ in their sensitivity to different chemotherapeutic agents.

## Data availability statement

The data presented in the study are deposited in the iProX repository, accession number PXD044500.

## Ethics statement

The studies involving human participants were reviewed and approved by Ethics Committee of First Hospital of Lanzhou University. The patients/participants provided their written informed consent to participate in this study.

## Author contributions

WZ and HZ are responsible for the design of the study and reviewed the manuscript. YD and XL contributed to the design of the study, collection, and interpretation of data, and drafting and revising the manuscript. WJ and SD participated in the collection and analysis of data. WL and HT are responsible for the collection and processing of samples. JY: Writing – review & editing, Methodology, Formal analysis, Software. All authors contributed to the article and approved the submitted version.
