# Supplementary material for: Integrated transcriptomics, proteomics and metabolomics-based analysis uncover TAM2-associated glycolysis and pyruvate metabolic remodeling in pancreatic cancer
Source: Front Immunol. 2023 Aug 17;14:1170223. doi: 10.3389/fimmu.2023.1170223 (PMC10470650; doi:10.3389/fimmu.2023.1170223)
Supplement: Supplementary file 1 [file DataSheet_1.docx]

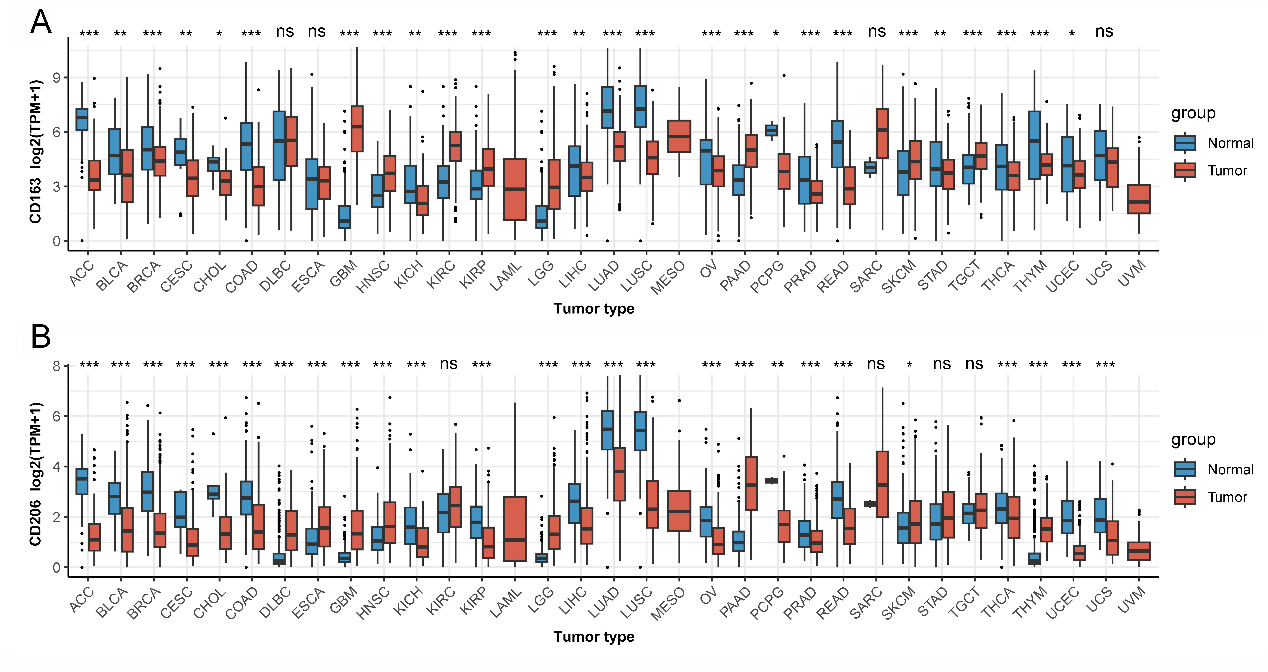


Figure S1: Pan-cancer analysis of the TAM2 cell markers CD163 (A) and CD206 (B) in 33 malignant tumor types of TCGA. TAM2, tumor-associated macrophage 2. *P < 0.05, **P < 0.01, ***P < 0.001.


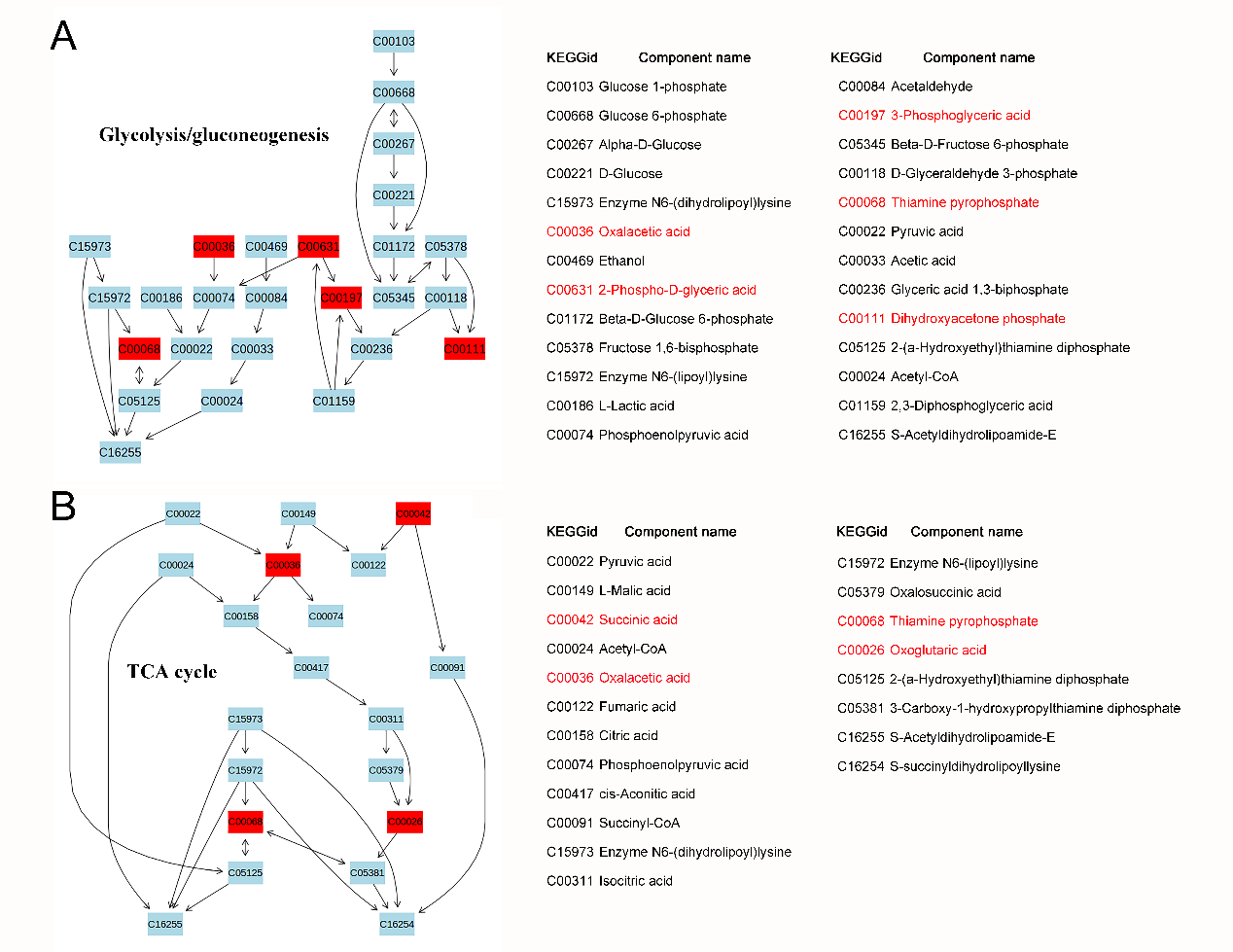


Figure S2: KEGG pathway diagrams for glycolysis (A) and TCA cycles (B).


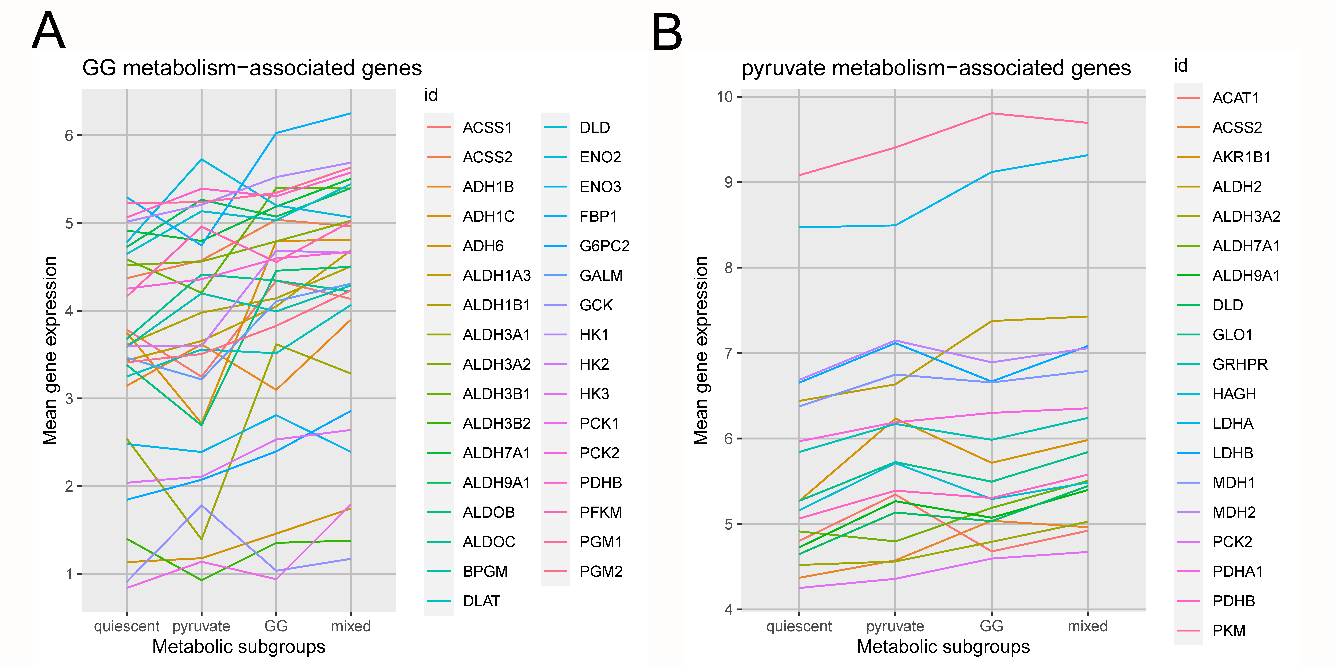


Figure S3: The average expression levels of GG (A) and pyruvate(B) metabolism-associated genes for each metabolic subtype.


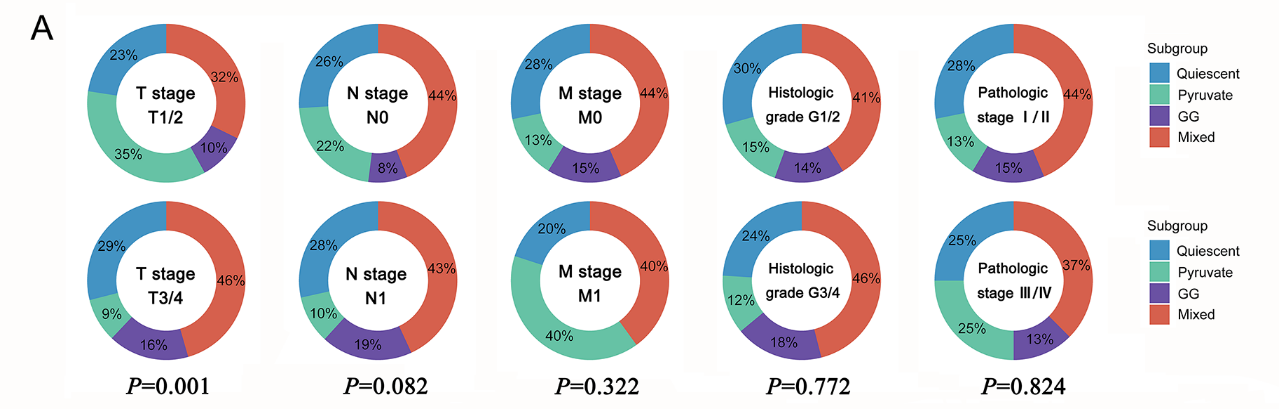


Figure S4: Percentage distribution of metabolic subtypes in TCGA-PAAD samples grouped by different clinical characteristics.
